# Chromophore supply modulates cone function and survival in retinitis pigmentosa mouse models

**DOI:** 10.1073/pnas.2217885120

**Published:** 2023-05-30

**Authors:** Yunlu Xue, Xiaomei Sun, Sean K. Wang, Gayle B. Collin, Vladimir J. Kefalov, Constance L. Cepko

**Affiliations:** ^a^Lingang Laboratory, 200031, Shanghai, China; ^b^Department of Genetics, Blavatnik Institute, Harvard Medical School, Boston, MA 02115; ^c^Department of Ophthalmology, Harvard Medical School, Boston, MA 02115; ^d^Department of Ophthalmology & Visual Sciences, Washington University School of Medicine, St. Louis, MO 63110; ^e^HHMI, Boston, MA 02115; ^f^The Jackson Laboratory, Bar Harbor, ME 04609

**Keywords:** retinitis pigmentosa, visual cycle, Alström syndrome, cone photoreceptors, retina

## Abstract

Retinitis pigmentosa (RP) is an ocular disease characterized by the loss of night vision, followed by the loss of daylight vision. Daylight vision is initiated in the retina by cone photoreceptors, which are gradually lost in RP, often as bystanders in a disease process that initiates in their neighboring rod photoreceptors. Using physiological assays, we investigated the timing of cone electroretinogram (ERG) decline in RP mouse models. A correlation between the time of loss of the cone ERG and the loss of rods was found. To investigate a potential role of the visual chromophore supply in this loss, mouse mutants with alterations in the regeneration of the retinal chromophore, 11-*cis* retinal, were examined. Reducing chromophore supply via mutations in *Rlbp1* or *Rpe65* resulted in greater cone function and survival in a RP mouse model. Conversely, overexpression of *Rpe65* and *Lrat*, genes that can drive the regeneration of the chromophore, led to greater cone degeneration. These data suggest that abnormally high chromophore supply to cones upon the loss of rods is toxic to cones, and that a potential therapy in at least some forms of RP is to slow the turnover and/or reduce the level of visual chromophore in the retina.

## Significance

Retinitis pigmentosa (RP) is a blinding disease affecting 2 million people worldwide. The night vision of RP patients is affected first due to the expression of a disease gene in rod photoreceptors. This loss of rod-mediated night vision is followed by a bystander effect on cone photoreceptors, which results in loss of daylight vision. The mechanism of the secondary cone degeneration is unclear. We used genetically modified mice and electrophysiology to explore this question. We found that slowing down the recycling of vitamin A derivatives for the regeneration of opsin, a GPCR, could protect RP cone function and extend their survival. These studies reveal a unique pathway for RP cone degeneration and suggest potential therapies that could benefit RP patients.

Retinitis pigmentosa (RP) is one of the most common inherited retinal degenerative diseases (IRDs), affecting 1 in 4,000 people worldwide (1). It is characterized by the loss of night vision, followed by the loss of color and daylight vision. The identification of many disease genes, which now number ~100 (https://sph.uth.edu/retnet/), revealed the molecular basis for the initial loss of night vision. Many of the disease genes are expressed only in rods, the photoreceptor type that detects light in dim light conditions. Cone photoreceptors, the photoreceptor type active in brighter light conditions, are then affected secondarily, generally after most of the rods in their neighborhood have died. The cause(s) of secondary cone death in RP is not clear, though studies have suggested that trophic factor loss, oxidative stress, metabolic changes, and immune responses contribute to the loss of cone function and survival (see review ref. 2). Support for these hypotheses comes from therapies that address some of these problems, as they have led to increased cone survival (3–17). Such therapies also have led to a retention of cone-mediated vision, as reflected in measurements made by the optomotor behavioral test. This test measures an animal’s response to moving bars of different sizes to determine its ability to detect different spatial frequencies. This is a sensitive assay that can show positive results even when only a small fraction of light-responsive cones remains.

Most studies utilizing gene therapy to prolong cone survival have not directly assayed cone physiology (10, 18). Similarly, studies of untreated RP mouse strains have not characterized cone physiology over time until recently with one RP strain (19), and several studies that did carry out such assessments have left some open questions that we wished to address. For example, following gene therapy aimed at improving cone survival and function in RP mice, electrophysiology measurements were made at a stage before all of the rods were gone (3). Any observed improvement in cone function could thus be due to effects on rods rather than direct effects on cones. Our own experiments aimed at improving cone survival often led to improved optomotor responses. However, we were unable to show improved cone function using electroretinograms (ERGs), a more direct assay of cone physiology (11, 14).

To address the issues mentioned above, we set out to characterize the cone ERG during photoreceptor degeneration in RP mouse models and to determine treatments that might improve it. We confirmed that the cone ERG became undetectable at a stage that correlated with the near-complete loss of rods. We further investigated whether this might be due to the loss of rod function, but found that it was due to the loss of the rods themselves. Notably, suppressing the recycling of the visual chromophore for phototransduction was found to preserve the cone ERG, improve cone opsin expression and localization, and extend cone survival after the rods died in RP mice. This leads to a model wherein RP cones are poisoned from excessive chromophore that reaches a toxic threshold when the last few rods die.

## Results

### ERG Measurements of the RP Cone Pathway during the Period of Rod Death.

We first examined the literature characterizing the ERG response in several commonly used RP mouse strains. As the cone ERG a-wave, which measures cone function, is too small to measure, even in wild-type eyes, studies of the cone ERG a-wave during the disease process in vivo have not been reported. Due to this difficulty, we focused on the cone ERG of the b-wave, which originates from cone ON-bipolar cells that are immediately downstream of cone photoreceptors. We examined this response in several mouse models of RP. The *Rho^tm1Phm^* knockout model, also known as *Rho^−/−^,* has a complete loss of rhodopsin. In this strain, ERG b-wave signals were detectable until postnatal day 80 to 90 (P80 to P90) (20, 21), the age when rods are almost completely gone (17, 22). We concluded that these ERG responses must have originated from the cone pathway, as rod phototransduction in *Rho^−/−^* mice should be absent due to the lack of rhodopsin. In the *rd10* strain, which carries a missense mutation in the *Pde6b* gene that is critical for rod phototransduction, a cone ERG b-wave was detected at P18-30 but lost by P50-63 when rods were no longer present (23). In addition, no cone ERG response was reported in the untreated *rd1* strain (23, 24), which carries a null mutation in the *Pde6b* gene, leading to fast degeneration of rods. Absence of an ERG in this strain was likely due to the complete loss of rods by ~1 mo of age, when the ERG was first measured (24). Together, these results suggest that the loss of cone ERG signals might be correlated with the loss of RP rods.

**Fig. 1.**
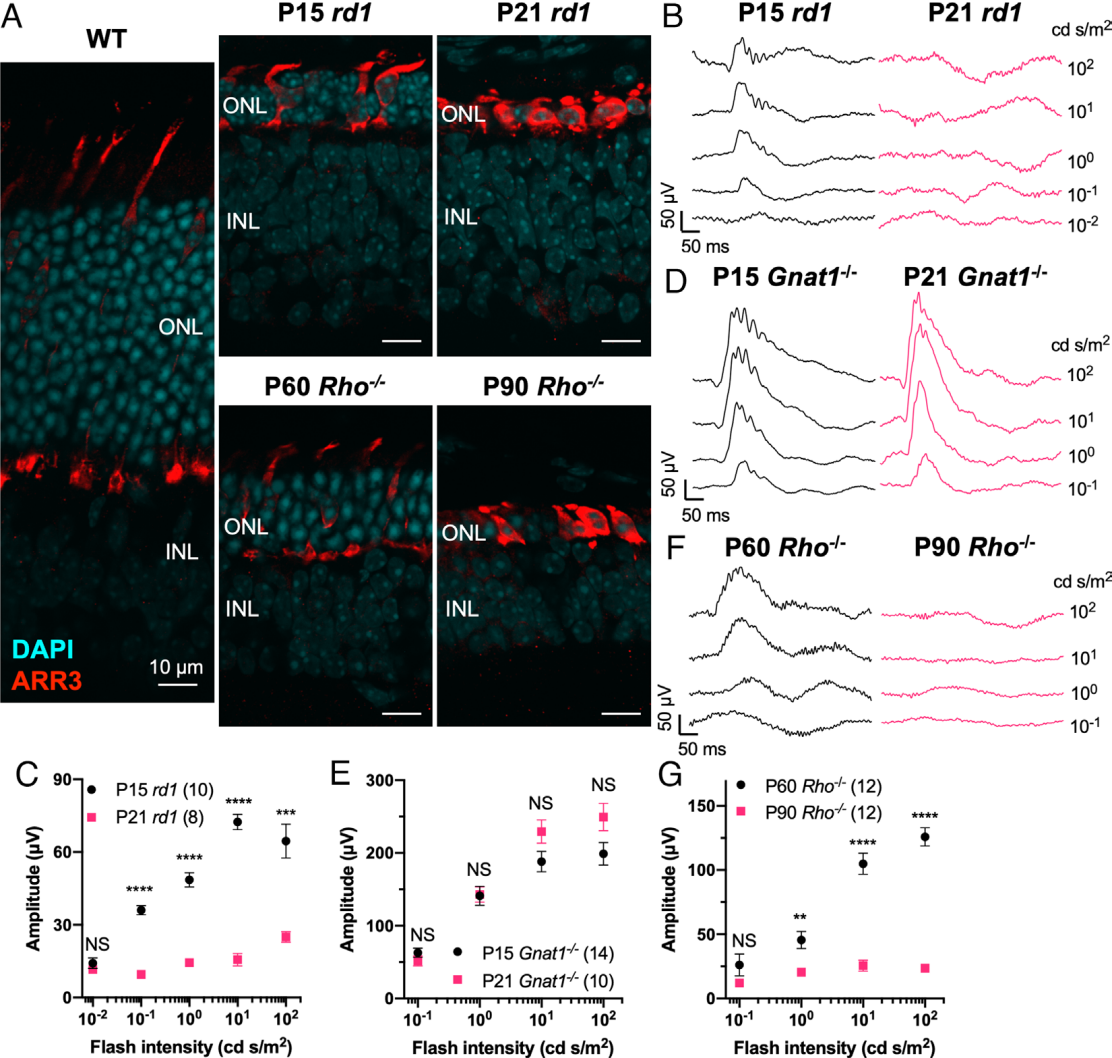
Characterization of histology and electroretinography (ERG) of RP eyes. (*A*) Images from immunohistochemistry carried out on postnatal day 39 (P39) wildtype (WT), P15 *rd1*, P21 *rd1*, P60 Rho-/-, and P90 *Rho*-/- retinal cross sections stained with DAPI (cyan), and ARR3 (red). ONL: outer nuclear layer; INL: inner nuclear layer. N = 5. (*B*) Representative dark-adapted ERG traces from P15 and P21 *rd1* eyes. Flash intensities eliciting traces are labeled on the right side in cd s/m^2^. (*C*) Ensemble-averaged dark-adapted ERG b-wave amplitude from P15 and P21 *rd1* eyes (same as in *B*). (*D*) Representative dark-adapted ERG traces from P15 and P21 *Gnat1^−/−^* eyes. (*E*) Ensemble-averaged dark-adapted ERG b-wave amplitude from P15 and P21 *Gnat1^−/−^* eyes (same as in *D*). (*F*) Representative dark-adapted ERG traces from P60 and P90 *Rho^−/−^* eyes. (*G*) Ensemble-averaged dark-adapted ERG b-wave amplitude from P60 and P90 *Rho^−/−^* eyes (same as in *F*). Error bar: SEM. NS: not significant; *P* > 0.05, **P < 0.01, ***P < 0.001, ****P < or <<0.0001. The number in the round brackets “()” indicates the number of eyes within each group.

To further examine whether the absence of a cone ERG correlated with rod loss in the *rd1* retina, we first tested cone function at P15 in *rd1*, a stage where 2-3 rows of rod nuclei would have been present in the outer nuclear layer (ONL) (Fig. 1*A*). ERG responses were observed in dark-adapted P15 *rd1* eyes (Fig. 1 *B* and *C*). In contrast, the ERG recordings of *rd1* mice at P21, a stage where almost all rods would have been lost (17, 23, 25), showed barely detectable signals (Fig. 1 *A*–*C*). Because the waveform and kinetics of the P15 *rd1* dark–adapted ERG looked similar to a pure cone pathway response (i.e., no a-wave, emergence of the b-wave ~0.1 cd s/m^2^), we wondered whether PDE6B deletion alone abolished phototransduction in rods, leaving only the signals from cones. In keeping with this, ERG responses have been observed in *Rho*^−/−^ mice, where rod phototransduction is absent but cones are functional and persist until the rods die (20).

To further examine whether the loss of rods or the absence of rod phototransduction was responsible for the differences in the *rd1* cone ERG at P15 vs. P21, we applied the same ERG protocol with transducin-α subunit-deficient mice (*Gnat1^tm1Clma^*, also referred as *Gnat1*^−/−^). These mice have a normal number and morphology of rods, but do not respond to light (26). Robust cone ERG signals were observed in *Gnat1*^−/−^ eyes at both P15 and P21 (Fig. 1 *D* and *E*), confirming that the loss of rod phototransduction is not the cause of RP cone ERG loss. This finding is also consistent with the observation of persistent ERG responses in adult *Gnat1*^−/−^ eyes in multiple previous studies (26–33).

To further investigate the correlation between RP rod number and RP cone ERG, ERG measurements were made using *Rho*^−/−^ mice at flash intensities spanning 4 log-units, which cover a brighter range than the previous longitudinal studies conducted at only one flash intensity (20, 21). In the retinas of *Rho*^−/−^ mice, ~3 to 4 rows of rod nuclei were present in the ONL at P60, while all rods were gone by P90 (Fig. 1*A*). ERG measurements at these ages showed a similar correlation between the presence of rod nuclei and the cone ERG (Fig. 1 *F* and *G*), consistent with the findings of the previous study (20, 21).

Although we wished to run the same ERG protocol on *rd10* mice, it was difficult to obtain reproducible data. Our protocol measures the dark-adapted cone responses without background light for strains that do not possess functional rods, such as *Rho*^−/−^, *rd1,* and *Gnat1*^−/−^. In *rd10*, rod phototransduction is active, while rod degeneration is geographically uneven and dependent upon light, which can vary within the mouse room. We thus did not carry out this ERG protocol in this strain.

In summary, the *rd1*, *Rho*^−/−^, and *Gnat1*^−/−^ results show that the disappearance of the cone ERG correlates with the loss of rods in the ONL, but not with the loss of rod phototransduction (see schematics in *SI Appendix*, Fig. S1).

### RP Cone ERG Signals Are Retained in Mice with *Rlbp1* Deficiency.

**Fig. 2.**
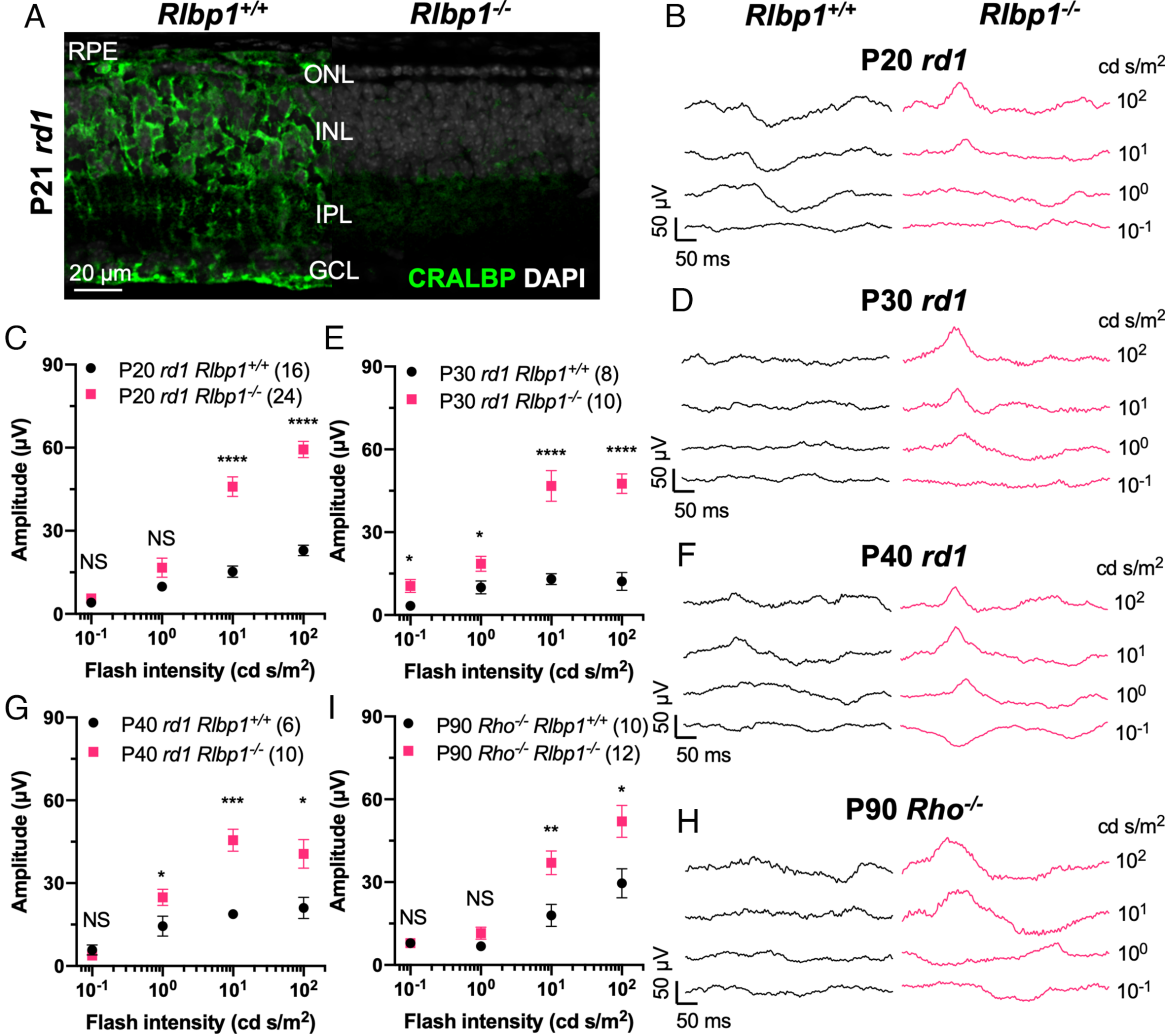
Effects of *Rlbp1* deficiency on RP mouse ERG. (*A*) Images from immunohistochemistry carried out on P21 *rd1*;*Rlbp1*^+/+^ and *rd1*;*Rlbp1*^−/−^ retinal cross-sections stained with DAPI (gray) and CRALBP (green). RPE: retinal pigmented epithelium; IPL: inner plexiform layer; GCL: ganglion cell layer. N = 3. (*B*) Representative dark-adapted ERG traces from P20 *rd1*;*Rlbp1*^+/+^ and *rd1*;*Rlbp1*^−/−^ eyes. (*C*) Ensemble-averaged dark-adapted ERG b-wave amplitude from P20 *rd1*;*Rlbp1*^+/+^ and *rd1*;*Rlbp1*^−/−^ eyes (same as in *B*). (*D*) Representative dark-adapted ERG traces from P30 *rd1*;*Rlbp1*^+/+^ and *rd1*;*Rlbp1*^−/−^ eyes. (*E*) Ensemble-averaged dark-adapted ERG b-wave amplitude from P30 *rd1*;*Rlbp1*^+/+^ and *rd1*;*Rlbp1*^−/−^ eyes (same as in *E*). (*F*) Representative dark-adapted ERG traces from P40 *rd1*;*Rlbp1*^+/+^ and *rd1*;*Rlbp1*^−/−^ eyes. (*G*) Ensemble-averaged dark-adapted ERG b-wave amplitude from P40 *rd1*;*Rlbp1*^+/+^ and *rd1*;*Rlbp1*^−/−^ eyes (same as in *F*). (*H*) Representative dark-adapted ERG traces from P90 *Rho*^−/−^;*Rlbp1*^+/+^ and *Rho*^−/−^;*Rlbp1*^−/−^ eyes. (*I*) Ensemble-averaged dark-adapted ERG b-wave amplitude from P90 *Rho*^−/−^;*Rlbp1*^+/+^ and *Rho*^−/−^;*Rlbp1*^−/−^ eyes (same as in *H*). Error bar: SEM. NS: not significant; *P* > 0.05, *P < 0.05, **P < 0.01, ***P < 0.001, ****P < or << 0.0001. The number in the round brackets “()” indicates the number of eyes within each group.

As the function of cones, not merely their survival, is critical for daylight vision, we asked how changes in the retina accompanying rod death might affect cone function. We postulated that the amount of the visual chromophore, and its derivatives, which might be buffered by rods, could affect cone function. In wild-type retinas, rods and cones might compete for the regenerated chromophore from the retinal pigmented epithelium (RPE). Following rod death, the abundant supply and/or accelerated turnover of retinoids for cones, especially retinaldehyde (i.e., retinal), might be harmful to cones. If true, slowing down the visual cycle, the process through which the visual chromophore is recycled, might preserve the function of RP cones. CRALBP is a carrier protein for 11-*cis* retinoids and is expressed in both RPE and Müller glial cells (34). We previously showed that CRALBP, encoded by the *Rlbp1* gene, is important for the chromophore supply to cones, and that the deletion of *Rlbp1* slows the dark adaptation of cones in mice (35). To investigate whether this gene might play a role in modulating the cone function of RP strains, we crossed the *Rlbp1*^−/−^ strain (also known as *Rlbp1^tm1Jsa^*) with the *rd1* strain. *Rlbp1*^−/−^;*rd1* double homozygous mice did not have any improvement in rod survival, as reflected by ONL thickness at P21 (Fig. 2*A*). However, when tested for cone function, ERG signals were observed at P20, P30, and P40. Age-matched *Rlbp1^+/+^;rd1* controls, which were derived from the same founders as those of *Rlbp1*^−/−^;*rd1* mice, had almost flat or noisy cone ERGs at these ages (Fig. 2 *B*–*G*). In addition, *Rlbp1*^−/−^;*Rho*^−/−^ double homozygous mice were generated and tested at P90. Improvement in ERG waveforms was observed in these mice, compared to the *Rlbp1*^+/+^;*Rho*^−/−^ controls derived from the same founders (Fig. 2 *H* and *I*).

### Cone Opsin Expression in *Rlbp1*-Deficient *rd1* Mice.

**Fig. 3.**
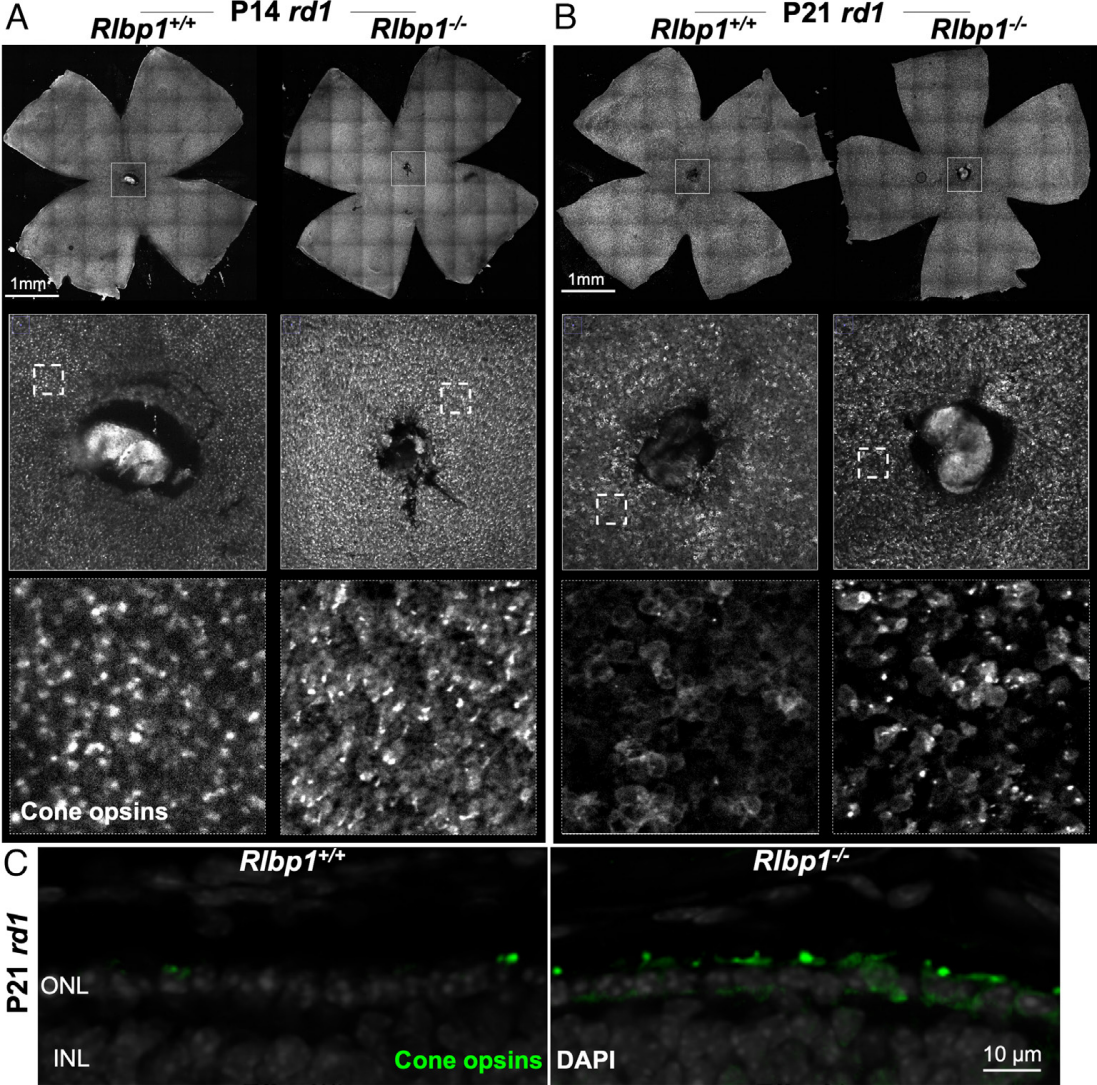
Effects of *Rlbp1* deficiency on RP cone opsin expression. (*A*) Images from immunohistochemistry carried out on P14 *rd1*;*Rlbp1*^+/+^ and *rd1*;*Rlbp1*^−/−^ flat-mounted retinas stained for cone opsins (a mixture of antibodies to OPN1SW and OPN1MW; white). Higher-magnification images of boxed regions are shown below. N = 8. (*B*) Images from immunohistochemistry carried out on P21 *rd1*;*Rlbp1*^+/+^ and *rd1*;*Rlbp1*^−/−^ flat-mounted retinas stained with anticone opsins (OPN1SW and OPN1MW; white). N = 8. (*C*) Images from immunohistochemistry carried out on P21 *rd1*;*Rlbp1*^+/+^ and *rd1*;*Rlbp1*^−/−^ retinal cross-sections stained with DAPI (gray) and anticone opsins (OPN1SW + OPN1MW; green). N = 4.

We next examined cone opsin expression during disease progression in *Rlbp1*-deficient mice. Antisera for the OPN1MW and OPN1SW proteins were combined and used to stain M-opsin and S-opsin together (*SI Appendix*, Fig. S2). The central retina of P14 *rd1* mice exhibited cone opsin “dots” resembling the degenerating cone outer segments (Fig. 3*A*), while the P21 *rd1* cones lacked such punctate structures and the cone opsins were mislocalized to the cell bodies (Fig. 3*B*). Similar to the P14 *Rlbp1*^+/+^*;rd1* mice, P14 *Rlbp1*^−/−^;*rd1* double homozygous cones had cone opsin–enriched dot structures (Fig. 3*A*), but unlike the P21 *Rlbp1*^+/+^ *rd1*;control, P21 *Rlbp1*^−/−^;*rd1* preserved more of these structures, as seen in both flat-mounts and cross-sections (Fig. 3 *B* and *C*).

### Cone Survival in *Rlbp1*-Deficient *rd1* Mice.

**Fig. 4.**
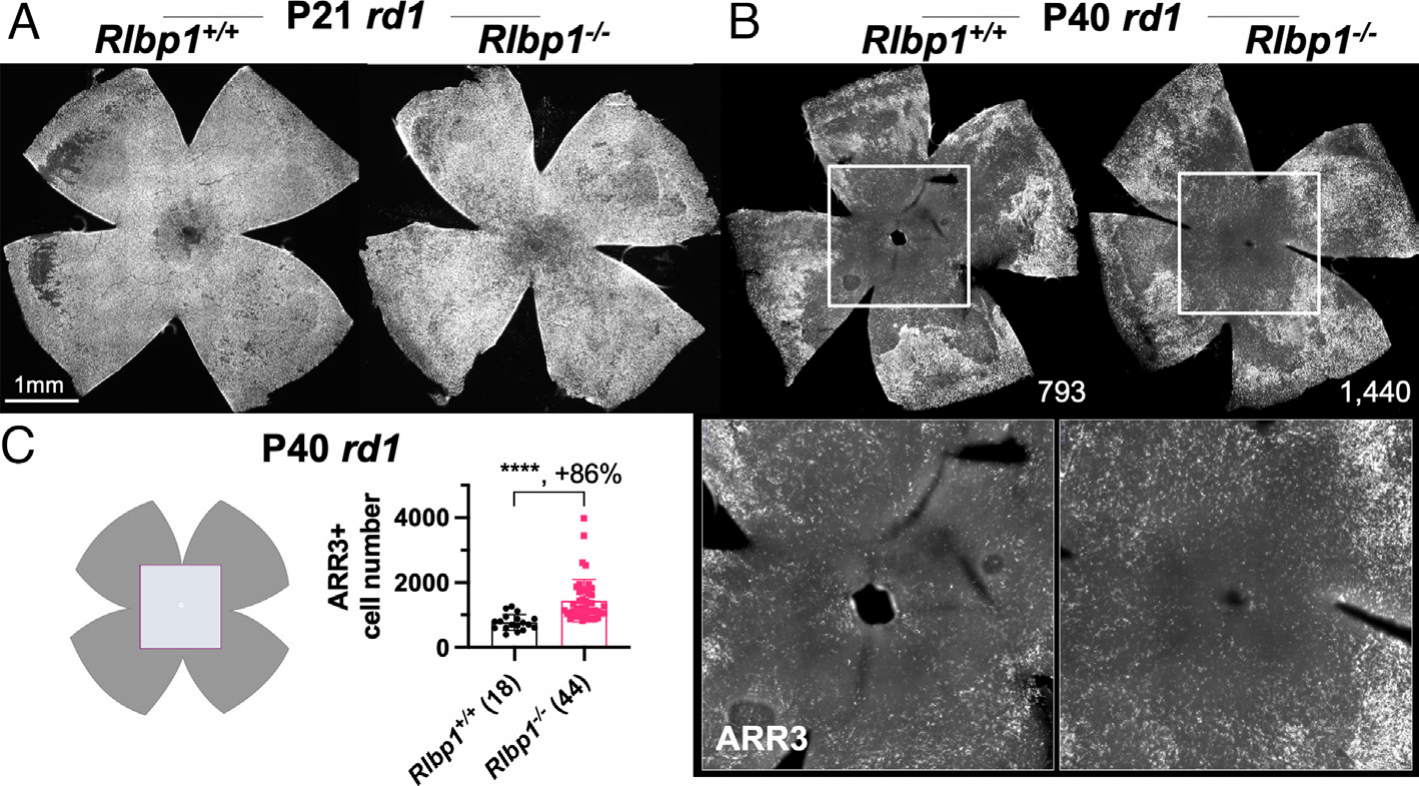
Effects of *Rlbp1* deficiency on RP cone survival. (*A*) Images from immunohistochemistry carried out on P21 *rd1*;*Rlbp1*^+/+^ and *rd1*;*Rlbp1*^−/−^ flat-mounted retinas stained with ARR3 (white). (*B*) Images from immunohistochemistry carried out on P40 *rd1*;*Rlbp1*^+/+^ and *rd1*;*Rlbp1*^−/−^ flat-mounted retinas stained for ARR3 (white). High-magnification images of boxed regions are shown below. (*C*) Quantification of ARR3-positive cones in the central retina for different groups (same as in *B*), using a previous published method (12). Error bar: SD. ****P < or <<0.0001. The number in the round brackets “()” indicates the number of retinas within each group.

To test whether RP cone survival would be improved with *Rlbp1* deficiency, the number of cones in *Rlbp1*^−/−^;*rd1* retinal flat-mounts was assayed using cone arrestin (ARR3) antibody staining. We first looked at P21, around the beginning of cone death, and observed ARR3 staining throughout the retina (Fig. 4*A*). Compared to the P21 retina, P40 ARR3 staining was dramatically decreased in the central retina of both *Rlbp1*^−/−^;*rd1* and *Rlbp1*^+/+^;*rd1* mice, suggesting severe cone degeneration and death from P21 to P40 (Fig. 4*B*), as has been reported for the *rd1* strain (36). Nonetheless, when the number of ARR3+ cells in P40 *rd1* retinas was quantified, there were significantly more cones (ARR3+ cells) in the central retina of *Rlbp1*^−/−^ *rd1* mice relative to the *Rlbp1*^+/+^ *rd1* mice (Fig. 4*C*).

### The Homozygous L450M Mutation in *Rpe65* Promotes Retention of the *rd1* Cone ERG and Survival.

**Fig. 5.**
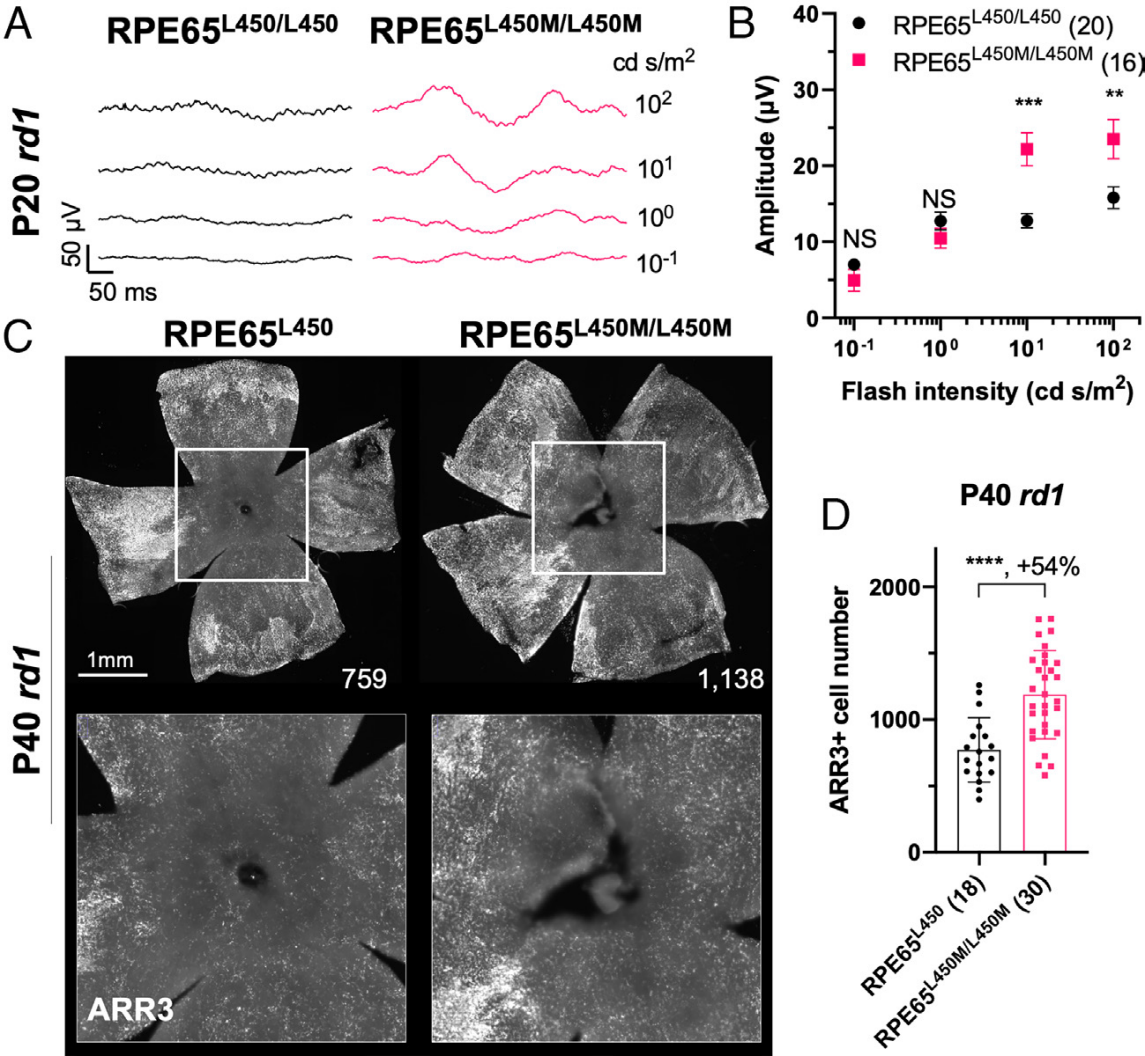
RP cone ERG and survival in the *Rpe65*-L450M variant. (*A*) Representative dark-adapted ERG traces from P20 homozygous *rd1*;*Rpe65^L450^* and homozygous *rd1*;*Rpe65^L450M^* eyes. (*B*) Ensemble-averaged dark-adapted ERG b-wave amplitude from P20 homozygous *rd1*;*Rpe65^L450^* and homozygous *rd1*;*Rpe65^L450M^* eyes (same as in *A*). Error bar: SEM. (*C*) Images from immunohistochemistry carried out on P40 *rd1*;*Rpe65^L450^* and homozygous *rd1*;*Rpe65^L450M^* flat-mounted retinas stained for ARR3 (white). Higher-magnification images of boxed regions are shown below. (*D*) Quantification of ARR3-positive cones in the central retina for different groups (same as in *C*). Error bar: SD. NS: not significant; *P* > 0.05, **P < 0.01, ***P < 0.001, ****P < or << 0.0001. The number in the round brackets “()” indicates the number of eyes within each group.

To further test the hypothesis that down-regulating the visual cycle benefits RP cone function and survival, a second strain, *Rpe65^L450M^*, was tested. RPE65 is expressed in the RPE and is a critical enzyme for the visual cycle through its activity in regenerating 11-*cis* retinoids (37). The L450M allele of RPE65 is naturally present in C57BL/6 strains and results in a slower RPE visual cycle and photoreceptor dark adaptation (28, 38), although to a lesser extent than *Rlbp1* deficiency. Accordingly, it does not cause cone degeneration in normal conditions. Mice carrying the *Rpe65^L450M^* allele were crossed to *rd1* and the ERG responses and cone numbers were characterized. Double homozygous *Rpe65^L450M^*;*rd1* mice showed an improved cone ERG at 10 and 100 cd s/m^2^, two of the four flash intensities tested, compared to the homozygous *Rpe65^L450^;rd1* controls that were derived from the same founders and carried the wild-type allele of *Rpe65* (Fig. 5 *A* and *B*). We also observed significantly more ARR3+ cones in the central retina of double homozygous *Rpe65^L450M^*;*rd1* mice than those of *Rpe65^L450^*;*rd1* control mice (Fig. 5 *C* and *D*).

### Ectopic RPE65 and LRAT Expression in Cones Reduces *rd1* Cone Survival.

**Fig. 6.**
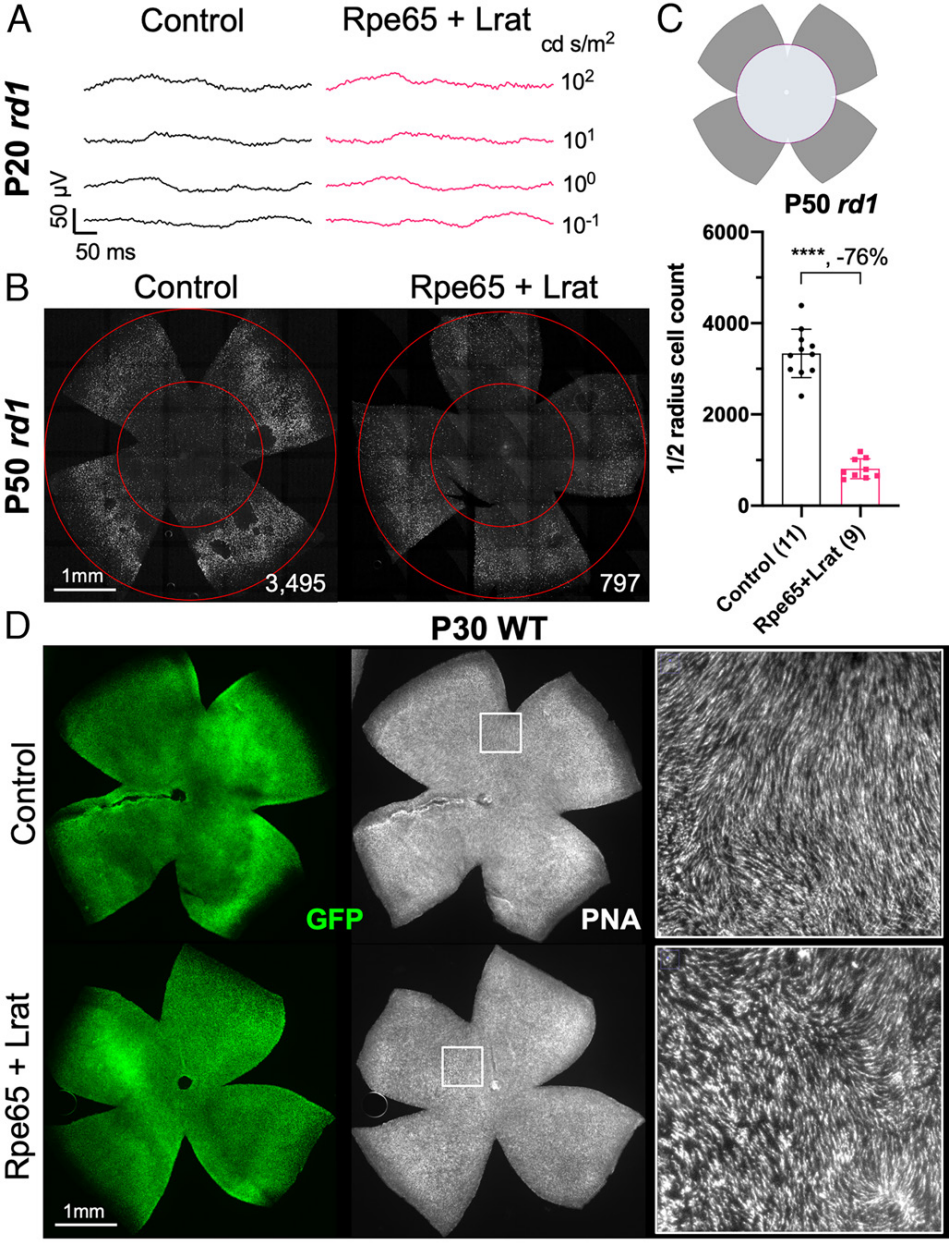
RP cone ERG and survival with ectopic expression of RPE65 and LRAT in cones. (*A*) Representative dark-adapted ERG traces from P20 *rd1* eyes P0-infected with adeno-associated viruses (AAVs) encoding RPE65, LRAT, and H2BGFP (AAV8-RedO-Rpe65, ≈1 × 10^9^ vg/eye, AAV8-RedO-Lrat, ≈1 × 10^9^ vg/eye, plus AAV8-RedO-H2BGFP, 2.5 × 10^8^ vg/eye) or control (AAV8-RedO-H2BGFP, 2.5 × 10^8^ vg/eye). N = 3. (*B*) Representative P50 *rd1* flat-mounted retinas after P0 infection with *Rpe65* + *Lrat*, or control (same as in *A*). (*C*) Quantification of H2BGFP-positive cones within the ½ radius of P50 *rd1* retinas transduced with *Rpe65* + *Lrat*, and control (same as in *B*). Error bar: SD. ****P < or << 0.0001. The number in the round brackets “()” indicates the number of eyes within each group. (*D*) Representative P30 WT flat-mounted retinas stained with PNA after P0 infection with *Rpe65* + *Lrat*, or control (same as in *A*). *Right* panels are high-magnification images from the regions indicated by boxes in the *Middle* panels.

As the loss of RPE65 activity or RLBP1 benefitted cone survival and function, it was of interest to determine whether the overexpression of related visual cycle genes might produce the opposite phenotype. All-*trans* retinol needs to travel from the photoreceptors to the RPE to be eventually converted to 11-*cis* retinal. RPE65 and LRAT are two critical enzymes that limit the conversion of all-*trans* retinol to 11-*cis* retinol in the RPE (39, 40), and 11-*cis* retinol can be further oxidized to 11-*cis* retinal by 11-*cis* RDH enzymes in the RPE (41). Subsequently, 11-*cis* retinal travels back to photoreceptors from the RPE to regenerate the visual pigments. Cones, but not rods, can directly use 11-*cis* retinol from Müller glial cells to regenerate the visual pigments (42, 43). If RPE65 and LRAT are ectopically expressed in cones, retinoids would not need to travel between cones and RPE/Müller glia, which might increase the chromophore turnover for cone opsin regeneration and their accumulation in cones. AAV vectors using a cone-specific promoter, RO1.7, which was made from a human red opsin promoter (44–46), were created to express RPE65 and LRAT in cones. They were injected along with a trace amount of AAV-RedO-H2BGFP for cone labeling. Injections were made into *rd1* retinas at P0, and ERGs were measured at P20. No ERG changes were noted at P20 between *Rpe65* + *Lrat*-transduced *rd1* retina and controls (i.e., H2BGFP only) (Fig. 6*A*). When retinas were examined for cone survival at P50, the number of cones was significantly lower in the AAV-*Rpe65* + *Lrat*-transduced retinas compared to the control at P50 (Fig. 6 *B* and *C*). In P30 wild-type retinas, AAV-*Rpe65* + *Lrat* infection resulted in no noticeable alteration in cone morphology or survival compared to the control, as probed by peanut agglutinin (PNA) or cone opsin staining (Fig. 6*D* and *SI Appendix*, Fig. S3).

### ALMS1 Disruption Reduces Cone Function and Accelerates Cone Dark Adaptation.

The above results suggested that an augmented visual cycle is harmful to cones, and we wondered whether any additional evidence from other models could support this notion. Searching the literature for other rod/cone disease genes, we took note of *Alms1*, the gene disrupted in the Alström syndrome. Alström syndrome is a multisystem disease that affects vision, hearing, heart function, as well as other systems. Loss of function of ALMS1 causes early-onset cone–rod dystrophy in humans (47). A previous characterization of *Alms1*^Gt(XH152)Byg^ gene trap mice (herein referred as *Alms1^−/−^*) showed decreased scotopic–photopic ERG amplitude and mislocalized rhodopsin in rods (48). As the mechanism for visual deterioration in ALMS1 is not clear, we set out to further investigate the physiological responses in *Alms1^−/^*^−^ mice by thoroughly examining the dynamics of cone dark adaptation.

**Fig. 7.**
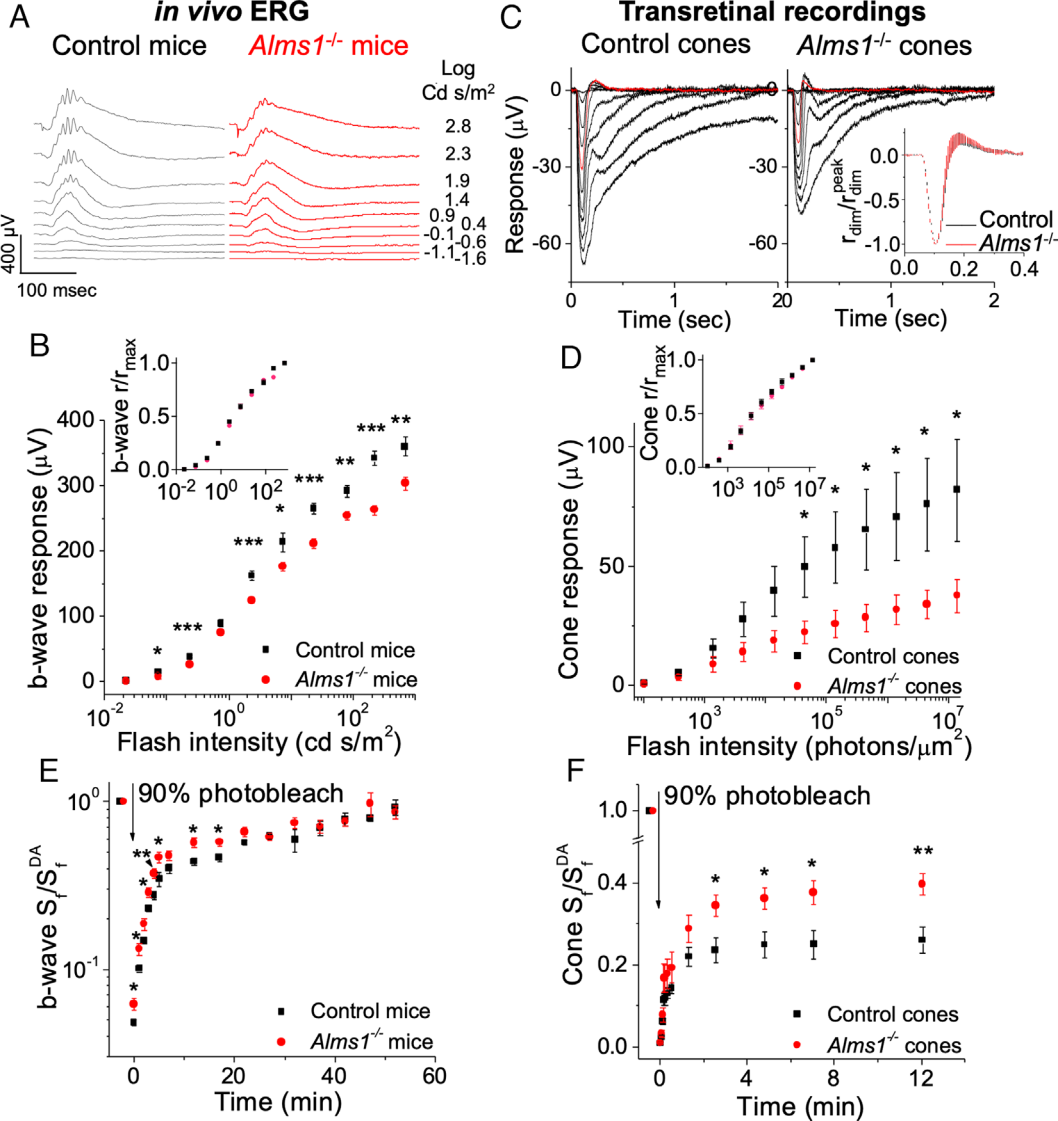
Cone pathway electrophysiology response and dark adaptation of six-week-old *Alms1*-mutant mice on *Gnat1*^−/−^ background. (*A*) Representative dark-adapted in vivo ERG traces from control (i.e., *Alms1*^+/+^*;Gnat1*^−/−^, same for all below) and *Alms1*^−/−^ (i.e., *Alms1*^−/−^*;Gnat1*^−/−^, same for all below) eyes. Flash intensities eliciting traces are labeled on the right side in log cd s/m^2^. (*B*) Averaged dark-adapted in vivo ERG cone b-wave intensity–response curves of control (n = 6) and mutant eyes (n = 6). *Inset*: the corresponding normalized intensity–response curves of the dark-adapted cone b-wave. (*C*) Representative ex vivo transretinal recording traces from control and *Alms1*^−/−^ retinas. The eliciting flash intensity of the red trace was 13,878 photons/μm^2^. Inset: averaged normalized dim-flash responses (elicited by 1,387 photons/μm^2^) of control (n = 6) and *Alms1*^−/−^ retina (n = 6). (*D*) Averaged intensity–response curve of control (n = 6) and *Alms1*^−/−^ groups (n = 8). *Inset*: normalized intensity–response curve of control (n = 6) and *Alms1 transformations*^−/−^ groups (n = 8). (*E*) In vivo ERG of cone b-wave sensitivity recovery following 90% photobleach, driven by both RPE and Müller glia visual cycles. The prebleach b-wave sensitivity, S_f_^DA^, was 161 ± 10 μV m^2^/cd s for control (n = 5) and 110 ± 7 μV m^2^/cd s for *Alms1*^−/−^ eyes (n = 6), **P < 0.01. (*F*) Transretinal recordings of cone sensitivity recovery following 90% photobleach, driven only by the Müller glia visual cycle. S_f_^DA^ was 9.2 ± 2.3 nV μm^2^/ph for control (n = 6) and 5.9 ± 1.9 nV μm^2^/ph for *Alms1*^−/−^ retinas (n = 8), *P* > 0.05. Error bar: SEM. Unlabeled: not significant; *P* > 0.05, *P < 0.05, **P < 0.01, ***P < 0.001.

To determine the consequence of *Alms1* disruption on cone function and dark adaptation, *Alms1*^−/−^ mice were crossed to mice with a null mutation in *Gnat1, Gnat1^irdr^* (49), herein referred to as *Gnat1^−/−^*. *Gnat1*^−/−^ mice have no rod function and thus ERGs or transretinal recordings would measure only cone function. The ERG responses from the double-mutant (*Alms1*^−/−^;*Gnat1*^−/−^) mice showed a significant reduction of cone b-wave amplitude at most of the flash intensities compared to the *Alms1*^+/+^;*Gnat1*^−/−^ mice that were derived from the same founders (Fig. 7 *A* and *B*). The normalized intensity–response curves for the *Alms1*^−/−^;*Gnat1*^−/−^ double mutant and the *Alms1*^+/+^;*Gnat1*^−/−^ eyes were comparable, suggesting that cone b-wave half-response intensity (I^b^_1/2_) was not affected by *Alms1* disruption (Fig. 7 *B*, *Inset*). Transretinal recordings (i.e., ex vivo ERG) were used to directly assess the cone function as it allows the isolation of the a-wave (Fig. 7*C*). Similar to the ERG b-wave results, the cone photoresponse amplitude in *Alms1*^−/−^;*Gnat1*^−/−^ mice was significantly reduced compared to *Alms1*^+/+^;*Gnat1*^−/−^ at most of the flash intensities (Fig. 7*D*), suggesting that *Alms1* disruption directly impairs cone function. The dim flash kinetics and the a-wave half-response intensity (I^a^_1/2_) of the *Alms1*^−/−^;*Gnat1*^−/−^ cones showed no difference from the *Alms1*^+/+^;*Gnat1*^−/−^ cones (Fig. 7 *C* and *D*, *Insets*), suggesting that the compromised cone function (i.e., dark current capacity) was not caused by issues in the amplification of the phototransduction cascade.

The *Alms1* gene trap disruption was investigated for effects on the kinetics of cone dark adaptation following exposure to bright light. Bright light bleaches most of the cone visual pigment, and the ERG can be used to measure the recovery of cone sensitivity following the bleach. Following bright light exposure, cone pigment regeneration and dark adaptation are driven by the chromophore, which is recycled by both the canonical visual cycle through the RPE and the cone-specific visual cycle through the Müller glial cells (50). The cone b-wave sensitivity (S^b^_f_) recovery was significantly increased in the *Alms1*^−/−^;*Gnat1*^−/−^ retina in the initial ~20 min following the bleach (Fig. 7*E*), implying an enhanced efficiency of chromophore turnover for cones in *Alms1*^−/−^ mice. The initial phase of cone recovery is driven by the retina visual cycle, whereas the later stage of cone dark adaptation is driven by the RPE visual cycle (28). To examine the operation of the cone-specific visual cycle directly, cone dark adaptation was measured in isolated retinas using transretinal recordings. Under these conditions, with the RPE removed, pigment regeneration is driven exclusively by the retina visual cycle (51). Consistent with the in vivo ERG results, the recovery of cone sensitivity in the isolated mutant retinas was significantly enhanced compared to control retinas in the 2 to 12 min postbleach (Fig. 7*F*). These results suggest that the cone-specific retinal visual cycle is enhanced in the *Alms1*^−/−^ retinas. The correlation between the enhanced chromophore recycling and the suppression of cone function in *Alms1* mutant mice is consistent with our observations in *rd1* and *Rho^−/−^* mice and suggests that a faster than normal chromophore recycling in cones could be detrimental to their function and long-term survival.

## Discussion

The goal of many gene therapies and small-molecule therapeutics for IRDs is to prolong cone survival and function. In our own studies using gene therapies aimed at a gene-agnostic approach, we found increased cone survival, but very little improvement in the cone ERG (11, 12, 14). Similarly, improvements in the cone ERG responses in other studies have been modest (3, 4, 13). This motivated an examination of cone function in untreated RP mice. Here, we report that cone ERG responses were nearly undetectable at an early stage of degeneration in mouse RP models (Fig. 1). This was surprising as the cones were all present, and were noticeably, but not yet drastically, altered in their morphology. By examining several strains of mutant mice, as well as the time course of the cone ERG loss, it is clear that the loss of the cone ERG is correlated with the loss of rods, rather than the absence of rod phototransduction, as expected. However, a recent study reported that the photopic light response from retinal ganglion cells persists in 7-mo-old *Cngb1^neo/neo^* mice, a RP strain in which most of the rods would have been lost by this age, as judged by the thickness of ONL (52). Another study, which employed patch-clamping, reported that the light response of cones persists in 9-wk-old *rd10* mice (19). While quantification of rods using a sensitive assay was carried out for *rd1* and *Rho^−/−^* in a previous study from our lab (17), a sensitive assay, such as RT-PCR, was not used to measure the remaining rods in these studies of *Cngb1^neo/neo^* (52) or *rd10* mice (19). Moreover, *Cngb1^neo/neo^* and *rd10* mice seem to be on the C57BL/6 background which carries the L450M allele of *Rpe65* (23, 53). Thus, it is not clear whether the two reports (19, 52) are in conflict with the findings presented here.

Our results suggest a few possibilities regarding the mechanism of cone dysfunction and degeneration in RP. A partial rescue of the cone ERG, and of cones themselves, was seen in mutants that have impaired chromophore transport or recycling. This led to the hypothesis that the amount of chromophore, and/or its derivatives, contributes to cone toxicity. There have been studies suggesting that a partially suppressed visual cycle, as defined by a slower turnover rate of chromophore, is protective to rods in certain conditions: *Rlbp1* deficiency protects rods from light-induced damage (34), as does the *Rpe65^L450M^* mutation (38). In both of these cases, there was a delay in the dark adaptation of rods and cones as well as slowed chromophore turnover (28, 34, 35, 38). Here, we found that these two genetic conditions also led to prolonged cone survival and function in RP mouse models (Figs. 2–5). The converse was also true, in that RP cone loss was exacerbated by misexpression, directly within cones, of the genes that can directly provide them with chromophore (Fig. 6). Interestingly, the cones in *Rho*^−/−^ mice have an acceleration in their dark adaptation, which reflects the speed of chromophore turnover in cones (54). This acceleration was seen before rods were gone, suggesting an enhanced access of recycled chromophore from the RPE in RP cones, possibly due to less competition from rods in the absence of rhodopsin.

All-*trans* retinal is produced from 11-*cis* retinal upon photon absorption. It is believed to be toxic to rods when it is present in abnormally high amounts. This notion is supported by a series of studies using *Abca4*^−/−^;*Rdh8*^−/−^ double homozygous mice, which lack the necessary machinery to clear all-*trans* retinal from rods (55–57). Although it has not been measured or reported, the total amount of retinoids in RP eyes might not be greatly reduced upon rod death, due to a lack of active clearance mechanisms for multiple forms of retinoids. As aldehydes are reactive electrophiles that can directly damage protein thiols and amines (see review ref. 58), an excessive amount of unbound and free retinaldehyde may directly harm cones. Rods might buffer the lipophilic retinoids by providing a local sink via their lipid-rich inner and outer segments. The ability of rods to buffer retinoids might regulate the amount of free 11-*cis* retinal available to cones, and/or the rods might absorb the all-*trans* retinal/retinol from cone phototransduction to retard their transport to the RPE/Müller glia for further recycling. In the *Rho*^−/−^ retina, before rods are gone, there may be an overall lower level of 11-*cis* retinal in dark-adapted retinas due to the lack of rhodopsin (59). However, the level of free unbound 11-*cis* retinal per cone could be higher in *Rho*^−/−^ than that in wild-type retinas. In fact, turning down the visual cycle alleviated cone dysfunction in the *Rho*^−/−^ strain when rods were gone (Figs. 1 and 2). These results suggest that it may not be the total amount, but the free, unbound form of 11-*cis* retinal and/or all-*trans* retinal that harms cones.

Beyond the mechanism discussed above, phototransduction itself might also contribute to the poor function and survival of RP cones. This possibility is supported by the death of rods in light damage models due, at least partially, to overactive phototransduction (60). Although light damage models are not equivalent to RP degeneration, a similar mechanism might affect RP cones, i.e., there might be a reduction in cone phototransduction if the supply of 11-*cis* retinal is reduced, leading to protection of cones. In addition, cones use NADPH to reduce all-*trans* retinal, which is generated from 11-*cis* retinal upon light activation, to all-*trans* retinol (61). A reduction of 11-*cis* retinal might reduce the consumption of NADPH in RP cones. The NADPH thus saved may provide the RP cones with more reductive power, which can be used to fight oxidative stress (62).

Our results may also help to explain a recent finding regarding a protective effect of retinoic acid (RA) on RP cone survival. We found that RA, produced by ALDH1A1 in peripheral Müller glia, protected peripheral cones in a RP mouse model (36). This enzyme might contribute to cone survival by changing the retinaldehyde to the acid form. It is clear that a transcriptional readout of RA is also involved as there is at least a partial protection of cones via an activated form of the RA receptor. However, both mechanisms may contribute to cone survival.

Recordings from the *Alms1*^−/−^ mice, a cone–rod dystrophy disease model, provide an additional set of data to consider. We show here that an *Alms1* disruption induces an acceleration of cone dark adaptation. This is not due to the loss of rods, since at the age tested (~6 wk old), the number of rods is similar between *Alms1*^−/−^ mutants and littermate controls (48). We noticed that *Alms1* RNA is enriched in cones relative to rods or Müller glia using a single-cell transcriptomic database (63). ALMS1 is believed to be part of the basal body and centrosome (64, 65), which are critical elements of the connecting cilium of photoreceptors (66). This structure is important for transport between the inner and outer segments of photoreceptors. The observation of an acceleration in dark adaptation suggests that *Alms1* disruption enhanced the rate of the cone-specific visual cycle (Fig. 7). The inner segments of cones get 11-*cis* retinol from Müller glial cells (42, 43, 51, 67). 11-*cis* retinol is transported to the outer segments, presumably through the connecting cilium, and becomes oxidized to 11-*cis* retinal in the outer segments (68). In addition, ex vivo experiments showed that 11-*cis* retinal could travel from inner segment to outer segment in cones, but not in rods (69), as may occur if a small pool of 11-*cis* retinal ever exists in the retina in vivo. We speculate that ALMS1 might serve to regulate this transport, ensuring that an excessive amount of 11-*cis* retinoids do not get transported to the cone’s outer segment. The loss or the dysfunction of ALMS1 might facilitate the transport of the 11-*cis* retinoids to the outer segment, thus enhancing cone chromophore turnover and contributing to the deterioration of cones in Alström syndrome.

In summary, our results suggest a unique mechanism by which the degeneration of rods could lead to the loss of cone function and survival by leading to abnormally high, and likely toxic, levels of visual chromophore in, or surrounding, cones.

## Materials and Methods

### Animals.

*rd1* mice carry the homozygous *Pde6b^rd1^* allele from the FVB strain. CD1 mice were purchased from Charles River Laboratories and used as the WT mice for histology. *Gnat1^tm1Clma^* and *Rho^tm1Phm^* mice were generated and gifted by Janis Lem (Tufts University, MA) (26, 70). *Rlbp1*^tm1Jsa^ mice were generated and gifted by John Saari (University of Washington, WA) (34). RPE65^L450M^ allele is on the C57BL/6J genetic background (38) and was purchased from The Jackson Laboratory. These mice were crossed to each other and genotyped by RT-PCR using primers (Transnetyx, Cordova, TN). FVB and *Rho^tm1Phm^* strains carry the RPE65^L450^ allele. *Alms1*^Gt(XH152)Byg^ mice (48) were crossed to *Gnat1^irdr^* mice (provided by Bo Chang, The Jackson Laboratory, ME), to generate double homozygous mice, and were genotyped in the Naggert Lab (The Jackson Laboratory, ME). All mice were raised in default 12:12 light–dark cycle of animal facilities. The ambient light at the bottom of the mouse cages varied between 10 and 50 lx.

### In Vivo ERG.

ERGs were recorded from mouse eyes using an Espion E3 System (Diagonsys LLC) or LKC® system as described previously (35, 71). Mice were dark adapted overnight and anesthetized with ketamine–xylazine cocktail (100/10 mg/kg) prior to the recordings. The pupils of the animals were dilated with 1% tropicamide eye-drops. Throughout the experiments, the body temperature was kept warm using a heating pad. ERG signals were picked up from the mouse cornea by electrodes immersed in phosphate-buffered saline (PBS). Before running the tests, the mice were stabilized for 15 min in darkness. For dark-adapted intensity–response experiments, the tests started from dim to bright flashes, and the averaged cone b-wave amplitude of multiple traces was measured at one intensity. Prior to the dark adaptation test, the dim flash response was recorded using several 0.238 cd s/m^2^ flashes for normalization purpose. An estimated 90% of the visual pigment was photobleached using a custom-made green LED light source. The sensitivity recovery was measured after the bleach and normalized to the prebleach dim flash response to construct the kinetics of dark adaptation as previously described (28).

### Ex Vivo Transretinal Recording.

*Alms1^−/−^*;*Gnat1*^−/−^ retinas were dissected from the eyes of overnight dark-adapted mice, which were euthanized by CO_2_, under an infrared light microscope in accordance with the institutional guidelines of Washington University. A custom-made recording chamber was used to mount the retina with the photoreceptor side facing the light source (72, 73). 37 °C Locke’s solution bubbled with 95% O_2_/5% CO_2_ was used to perfuse the retina in the chamber. The perfusion solution also contained 30 μM DL-AP4 to block the mGluR-mediated synaptic transmission, inhibiting the response from ON-bipolar cells (i.e., the b-wave of ERG). Prior to the testing flashes, the retina was stabilized for 15 min in the recording chamber in complete darkness. Responses from photoreceptors were induced by a 505-nm LED light. The parameters of flashes, such as intensity and duration, were fine-controlled through Plamp9 software (Molecular Devices). With light stimuli spanning ~5 log units, the transretinal recording signals of cones (i.e., the voltage change across the retina) were amplified, digitized, and recorded in a computer to construct the intensity–response curve of cones. For the dark adaptation test, a bright 3-s light was first delivered to the retina to bleach an estimated 90% of the visual pigment. Then, the recovery of photoresponses was recorded immediately using a pre-programmed protocol over a ~12-min period. To reconstruct the dark adaptation of the sensitivity, the recorded recovery was normalized to its prebleach level of dim flash response as previously described (35).

### Subretinal Injection of AAVs.

The AAVs were designed, prepared, and delivered to eyes as previously described (14). We used Gibson assembly to clone AAV-RO1.7-Rpe65 and AAV-RO1.7-Lrat. The cDNAs of mouse *Rpe65* (#EX-Mm35203-M02) and *Lrat* (#EX-Mm12130-M02) were purchased from GeneCopoeia (Rockville, MD). The vectors were packaged into the AAV8 capsid, produced following transfection of 293T cells, and concentrated using iodixanol gradients as previously described (4, 74). AAVs were mixed together and diluted with PBS before injection into the subretinal space of P0 mouse eyes as previously described (4, 75).

### Histology and Cone Quantification.

The frozen sections and flat mounts of retinas were collected as previously described (76). Two methods of cone quantification were performed, based on previously established methods: 1) cone arrestin staining, and counting with ImageJ (12). 2) Labeling with coinjected AAV-RedO-H2BGFP, and counted with a MATLAB script (14).

## Supplementary Material

Appendix 01 (PDF)

## Data Availability

All study data are included in the article and *SI Appendix*.
