# Supplementary material for: Chromophore supply modulates cone function and survival in retinitis pigmentosa mouse models
Source: Proc Natl Acad Sci U S A. 2023 May 30;120(23):e2217885120. doi: 10.1073/pnas.2217885120 (PMC10266038; doi:10.1073/pnas.2217885120)
Supplement: Supplementary file 1 — Appendix 01 (PDF) [file pnas.2217885120.sapp.pdf]

PNAS

Supporting Information for
Chromophore supply modulates cone function and survival in retinitis pigmentosa mouse models

Yunlu Xue[1,2,5*], Xiaomei Sun[1], Sean K. Wang[2,3], Gayle B. Collin[6], Vladimir J. Kefalov[4,5*], Constance L. Cepko[2,3*]

1. Lingang Laboratory, Shanghai, China, 200031
2. Departments of Genetics and Ophthalmology, Harvard Medical School, Boston, MA 02115
3. Howard Hughes Medical Institute, Boston, MA 02115
4. Current address: Gavin Herbert Eye Institute & Center for Translational Vision Research, University of California, Irvine, CA 92697
5. Department of Ophthalmology & Visual Sciences, Washington University School of Medicine, St. Louis, MO 63110
6. The Jackson Laboratory, Bar Harbor, ME 04609

*Yunlu Xue, Vladimir J. Kefalov, Constance L. Cepko.
Email: ylxue@lglab.ac.cn, vkefalov@uci.edu, or cepko@genetics.med.harvard.edu

This PDF file includes:

Figures S1 to S3
SI References

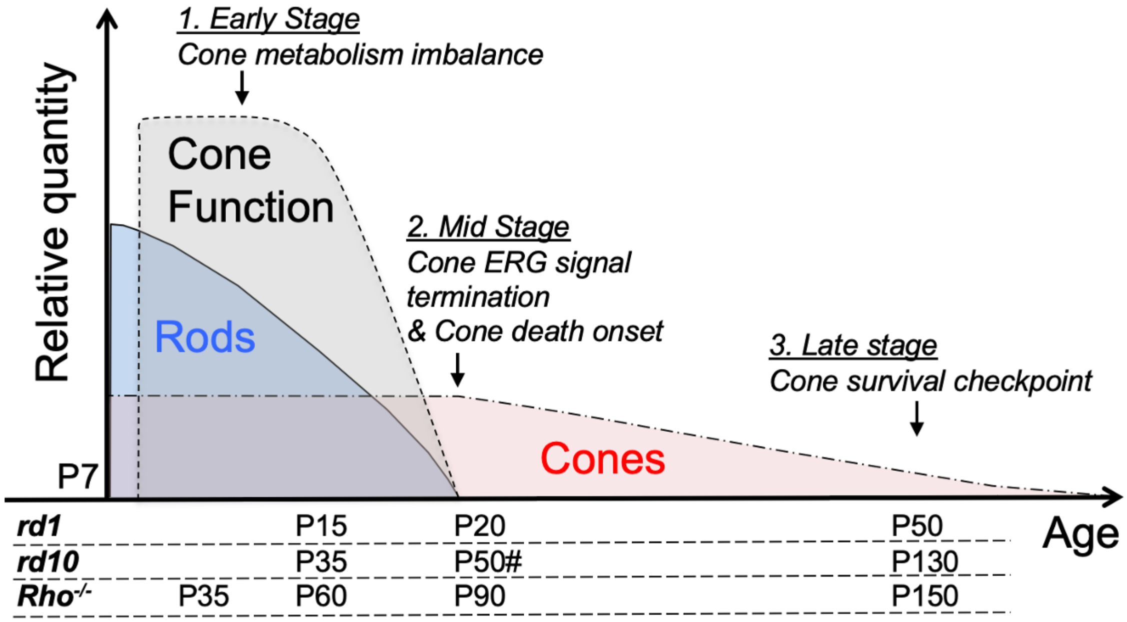

Fig. S1. Schematics of photoreceptor degeneration and cone ERG loss in retinitis pigmentosa (RP) mice. # rd10 mid stage varies due to light-dependent rod degeneration (1).

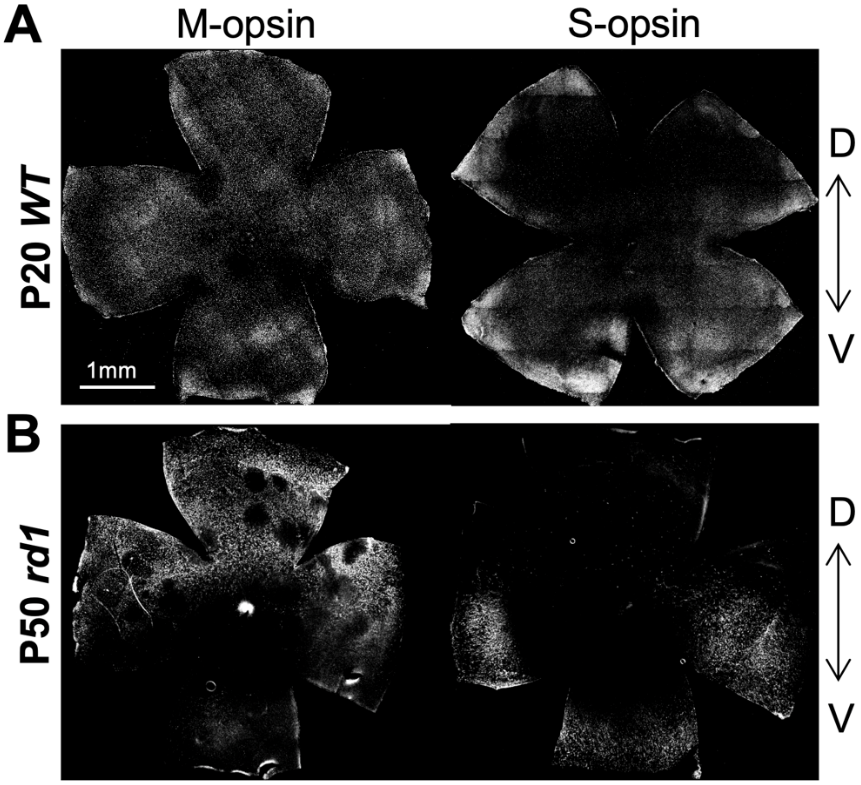

Fig. S2. Images from immunohistochemistry carried out on (**A**) P20 WT and (**B**) P50 rd1 flat-mounted retinas stained for cone M-opsin (OPN1MW) or S-opsin (OPN1SW). D: dorsal retina; V: ventral retina. N=4.

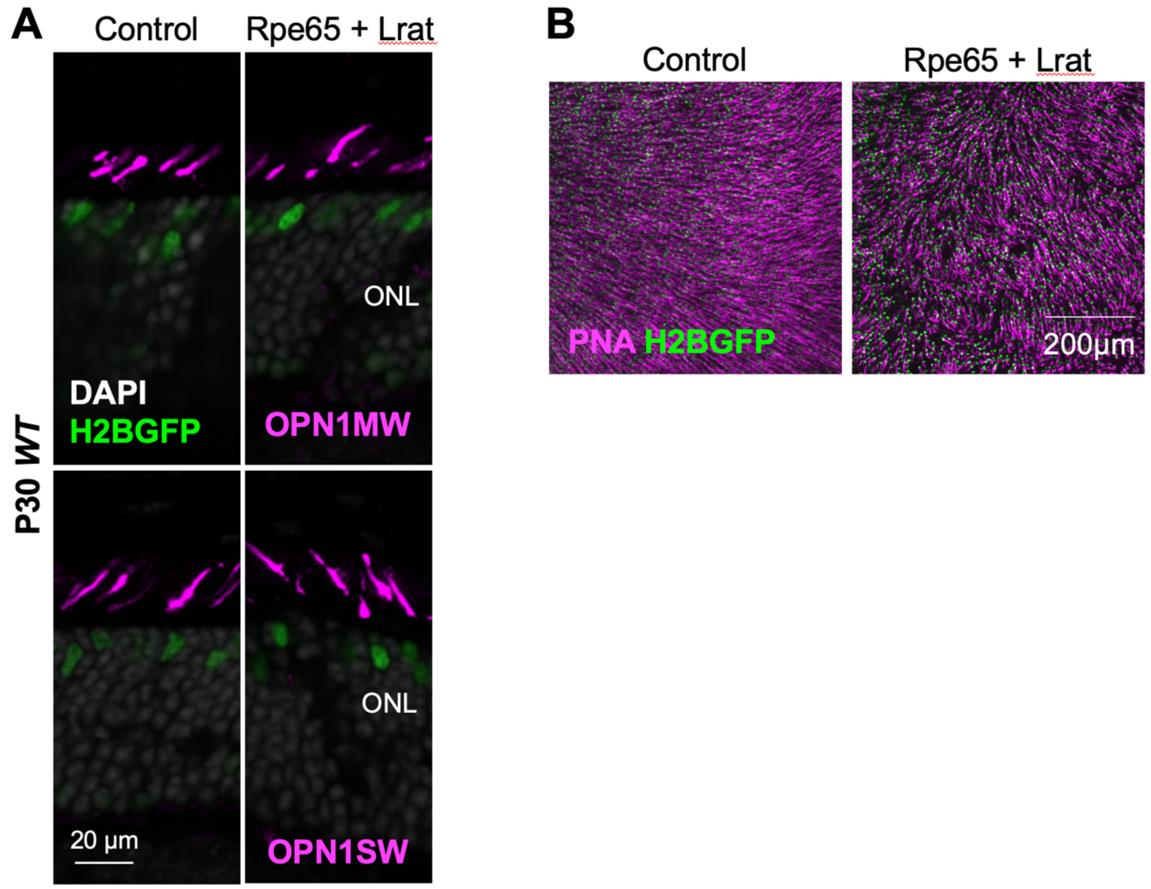

Fig. S3. WT cone morphology of retinas transduced by ectopic RPE65 and LRAT. (**A**) Representative cross section images of P30 WT retina transduced by control, and *Rpe65* + *Lrat* (same as in **Figure 6**) stained for OPN1MW or OPN1SW. (**B**) Representative high-resolution images of P30 WT flat-mounted retinas transduced by control and *Rpe65* + *Lrat* stained for PNA.

SI References

1. B. Chang, *et al.*, Two mouse retinal degenerations caused by missense mutations in the β-subunit of rod cGMP phosphodiesterase gene. *Vision Res.* **47**, 624–633 (2007).
